# Improved YOLOv7 enhances identification of *Hylurgus ligniperda* in traps

**DOI:** 10.3389/fpls.2026.1740965

**Published:** 2026-03-17

**Authors:** Zhengyi Li, Xiahui Zhang, Jing Tao

**Affiliations:** Beijing Key Laboratory for Forest Pest Control, Beijing Forestry University, Beijing, China

**Keywords:** deep learning, *Hylurgus ligniperda*, pest detection, YOLOv7, YOLOv8

## Abstract

The red-haired pine bark beetle, *Hylurgus ligniperda* Fabricius, is an internationally significant forest quarantine pest that poses a threat to coniferous trees in the coastal areas of Shandong, China. Monitoring its infestation is crucial in forest pest management, allowing for timely detection, early intervention, and prevention of further spread. Conventional manual identification methods are insufficient for modern surveillance of the *H. ligniperda*. To address this challenge, an improved YOLOv7 deep learning model is applied for identification. The aim is to automate, efficiently, and accurately identify and quantify the small, densely distributed, and variably posed *H. ligniperda* in the natural environment. The original backbone feature extraction network in YOLOv7 was replaced with the more lightweight and efficient EfficientNet Version 2 Small (EfficientNetV2-S) network to achieve model lightweighting while balancing speed and accuracy. Focal Loss was utilized as a loss function to mitigate the impact of class imbalance, balancing the ratio of positive and negative samples, thereby enhancing identification precision. Training on a dataset composed of pest images captured within traps demonstrated that the improved YOLOv7 achieved an impressive mean average precision (mAP) of 82.5%, a 4.4% improvement over the original YOLOv7. Comparative experiments with other models indicated superior performance of this enhanced model in the practical detection of *H. ligniperda.* This offers a viable solution for the precise identification of small pests and presents a practical application value for pest monitoring and early warning systems.

## 1 Introduction

Forest diseases and pests remain a major concern and challenge to the health and sustainable development of forests (Liu et al., 2022). *Hylurgus ligniperda* (Fabricius) (Coleoptera: Scolytidae) is a xylophagous pest causing damage to pine species (Lin et al., 2021). Identified as one of the most frequently intercepted forestry pests in Chinese port quarantine, *H. ligniperda* was first intercepted in imported Chilean logs in Tianjin, China, on April 14, 1986 (Wei and Shao, 1991). Its establishment and severe damage in Yantai and Weihai cities in Shandong Province were first discovered in 2020. Subsequently, its monitoring and trapping in Tai’ an City, Shandong Province, was carried out using plant-based attractants in 2019 (Ren et al., 2021). In recent years, *H. ligniperda* has been discovered in Qingdao, Weifang, and Rizhao in Shandong.

Biological invasions pose significant threats to agriculture, forestry, natural environments, and public health (Meyerson and Mooney, 2007). As a critical newly-invaded forestry pest in China, the primary monitoring method for *H. ligniperda*, involves deploying plant-based pheromone traps within forest ecosystems. However, this traditional method still requires periodic manual inspections for insect identification and counting, leading to issues of high labor intensity, costliness, time consumption, and low efficiency. Additionally, this subjective approach heavily relies on the expertise of insect classifiers (Sun et al., 2018).

With the emergence of machine learning methods, researchers have begun integrating them into insect detection. For instance, Wang et al. (2012) proposed a butterfly family identification approach based on color, morphology, and texture features. Similarly, Hassan et al. (2014) introduced a feature descriptor-based methodology for grasshopper and butterfly identification using color and shape characteristics. Yang et al. (2015) utilized a support vector machine-based method to identify insects with varying wing sizes. Despite the successes achieved by these methods, traditional machine learning approaches require manual feature design and extraction, exhibiting clear limitations and failing to fully meet the demands for fully automated monitoring of modern forestry pests. Moreover, the complexity of field environments complicates insect detection (Rustia et al., 2018; Liu and Wang, 2021; Tang et al., 2023).

In recent years, driven by rapid advancements in computer science and technology, machine learning-based object detection algorithms and image recognition techniques have garnered widespread research attention and application (Liu et al., 2020). Interdisciplinary integration have enabled the extensive application of deep learning methods in the detection of agricultural and forestry crop diseases and pests, significantly enhancing the accuracy and efficiency of pest detection and offering novel solutions for protecting forest ecosystems and pest control (Hossain et al., 2018; Júnior and Rieder, 2020). For instance, Cheng et al. (2017) proposed a pest classification method based on deep residual networks, achieving an accuracy of 98.67%. Tang et al. (2021) introduced a real-time agricultural pest detection method, Pest-YOLO, based on an improved YOLOv4, with an average precision of 71.6%, enabling real-time pest detection. Moreover, Ye et al. (2022) designed a model based on multiscale attention-UNet (MA-UNet) for large-scale satellite image monitoring, achieving a recall rate of 57.38% in detecting forest pest and disease areas, thereby improving forest management efficiency. Deep learning techniques have also been applied in developing image recognition applications. For instance, Chen et al. (2021) developed a smartphone application for pest detection, capable of assisting users in identifying potential pests on plants and providing relevant control suggestions. Despite the significant breakthroughs achieved by deep learning in these areas, there remains considerable room for improvement in the accuracy of real-time detection in wild forest environments (Yang et al., 2024; Miao et al., 2025). Deep learning object detection network models that have made significant progress include Faster R-CNN (Faster region with the convolutional neural network) (Ren et al., 2017), ResNet (Residual Network) (He et al., 2016), SSD (Single Shot Multibox Detector) (Wang et al., 2023) and YOLO (You Only Look Once) (Redmon et al., 2016; Zha et al., 2021; Zhang et al., 2025).

This study adopted an optimized detection scheme based on the deep learning model YOLOv7, which was aimed to maintain the lightweight characteristics of the model while enhancing monitoring accuracy and speed for automated detection of *H. ligniperda* in the natural environments (Terven et al., 2023). YOLOv7, as a real-time object detector, has demonstrated outstanding performance on large-scale image recognition datasets such as ImageNet (Lukas et al., 2022) and MS COCO (Lin et al., 2014), exhibiting high average precision. Compared to other traditional models, YOLOv7 exhibits superior real-time detection capabilities (Wang et al., 2022). To achieve this goal, we introduced EfficientNetV2-S, which exhibits excellent performance in image feature extraction and is lightweight, making it suitable for embedding in devices such as Raspberry Pi. Furthermore, we applied the Focal Loss approach to enhance the model’s ability to focus on learning and recognizing key features, thereby aiding faster convergence and more accurate localization and identification of small target samples, ultimately optimizing the model’s performance.

This research builds upon and extends previous studies conducted by our team, which primarily focused on using the unimproved YOLOv7 model to identify *H. ligniperda* in laboratory settings, achieving an mAP of 90.92%. However, in this study, we targeted scenes within traps in field environments, utilizing the improved YOLOv7 model for identification. This new direction aligns more closely with practical application needs and further validates and extends the laboratory research results.

The contributions of this paper are summarized as follows:

1. Adoption of EfficientNetV2-S as the backbone network addressed information loss when handling small target features, achieving model lightweighting while maintaining recognition accuracy and precision.
2. Application of Focal Loss enhanced the model’s focus on learning and recognizing key features, facilitating faster convergence and more accurate localization and identification of small target samples, thereby optimizing the model’s performance.
3. Experimental results demonstrated that the improved YOLOv7 achieved an mAP of 82.5% and a recall rate of 80.6%, surpassing other mainstream methods.

## 2 Materials and methods

### 2.1 Experimental data collection

The experimental data were collected from Yantai, Weifang, Rizhao, Weihai, and Qingdao in Shandong Province. The focus of detection comprised xylophagous pests captured using plant-based pheromone traps, specifically *H. ligniperda*. Images within the traps were captured using a Teslong camera with pixel parameters set at 1920 × 1080. A total of 2,652 images were captured, and the dataset was randomly categorized into training, validation, and test sets, accounting for 70%, 20%, and 10% of the entire dataset, respectively.

### 2.2 Image processing and data augmentation

**Figure 1:**
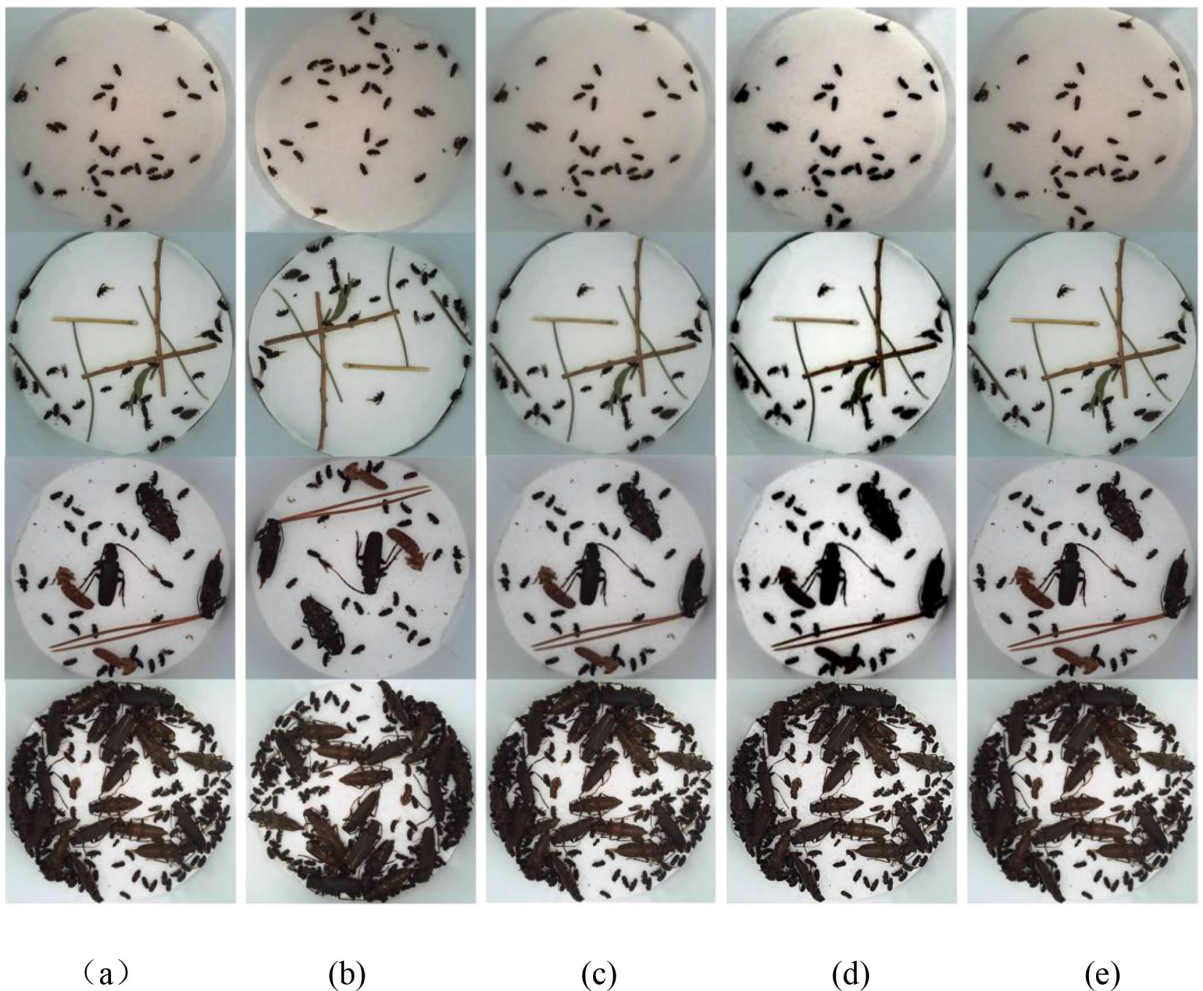
The visual effects of various data augmentation methods. **(a–e)** represent the type of each column

For high-resolution input images, deep learning models demand more resources and bear heavier burdens. Therefore, we processed the original images by cropping and normalization using the MATLAB Vision Library, unifying the image resolution to 640 × 640. Subsequently, we employed the Labellmg annotation tool to categorically label dataset images, preserving them in the PASCAL VOC dataset format. Finally, the annotated training set was loaded into the model for training. This sequence of processing aimed to optimize the input data, enabling the model to effectively learn and recognize the target object (*H. ligniperda)*. Trap interiors in natural environments can be obscured by fallen branches, leaves, and variations in lighting conditions. These factors might reduce the recognition accuracy and performance of deep learning models (Ding and Taylor, 2016). Applying such datasets directly to identification models could result in less precise recognition of the *H. ligniperda* in specific scenarios, leading to poorer performance that fails to meet real-time monitoring requirements. Therefore, specific data pre-processing and augmentation methods are necessary to enhance the model’s capability to identify complex backgrounds and unique circumstances, ultimately improving overall recognition accuracy and performance. In this study, various data augmentation methods were employed to introduce random transformations to the training images, enhancing data diversity. These methods included random flips (horizontal and vertical), Gaussian blur, random cropping, random padding, random contrast adjustment, cutout, among others (Devries and Taylor, 2017). Different augmentation operations were randomly applied in each training batch to strengthen the model’s generalization capability. These strategies contributed to enhancing the model’s ability to identify *H. ligniperda* in special environmental conditions, thereby improving overall performance and robustness. Data augmentation results are illustrated in **Figure 1**. (a) represents the original image; (b) is the image after flipping; (c) is the image after Gaussian blur processing; (d) is the image after random contrast adjustment; and (e) is the image after random cropping.

### 2.3 The structure of the YOLOv7 model

Field monitoring of *H. ligniperda*, highly requires real-time monitoring and detection accuracy. YOLOv7, a robust object detection model, meet this requirement by showcasing outstanding performance in real-time applications. Its support for multi-scale detection enable its effective handling of targets of various sizes and scales, making it suitable for recognizing *H. ligniperda* within trap images captured in diverse natural environments. The YOLOv7 network architecture primarily comprises three parts: the Backbone network, Neck network, and Prediction network:

1. The Backbone network structure, building on YOLOv5, incorporates the E-ELAN and MPConv structures.
2. The Neck network structure mainly includes the SPPCSPC module and an optimized PAN module, allowing for the fusion of feature information among different feature layers. This fusion outputs to the Prediction section to generate detection boxes of different scales.
3. The Prediction network structure utilizes a loss function similar to YOLOv5, incorporates RepVGG-style modifications to its network structure, employs auxiliary head training, and adopts corresponding positive and negative sample matching strategies.

### 2.4 Improved YOLOv7 detection model

**Figure 2:**
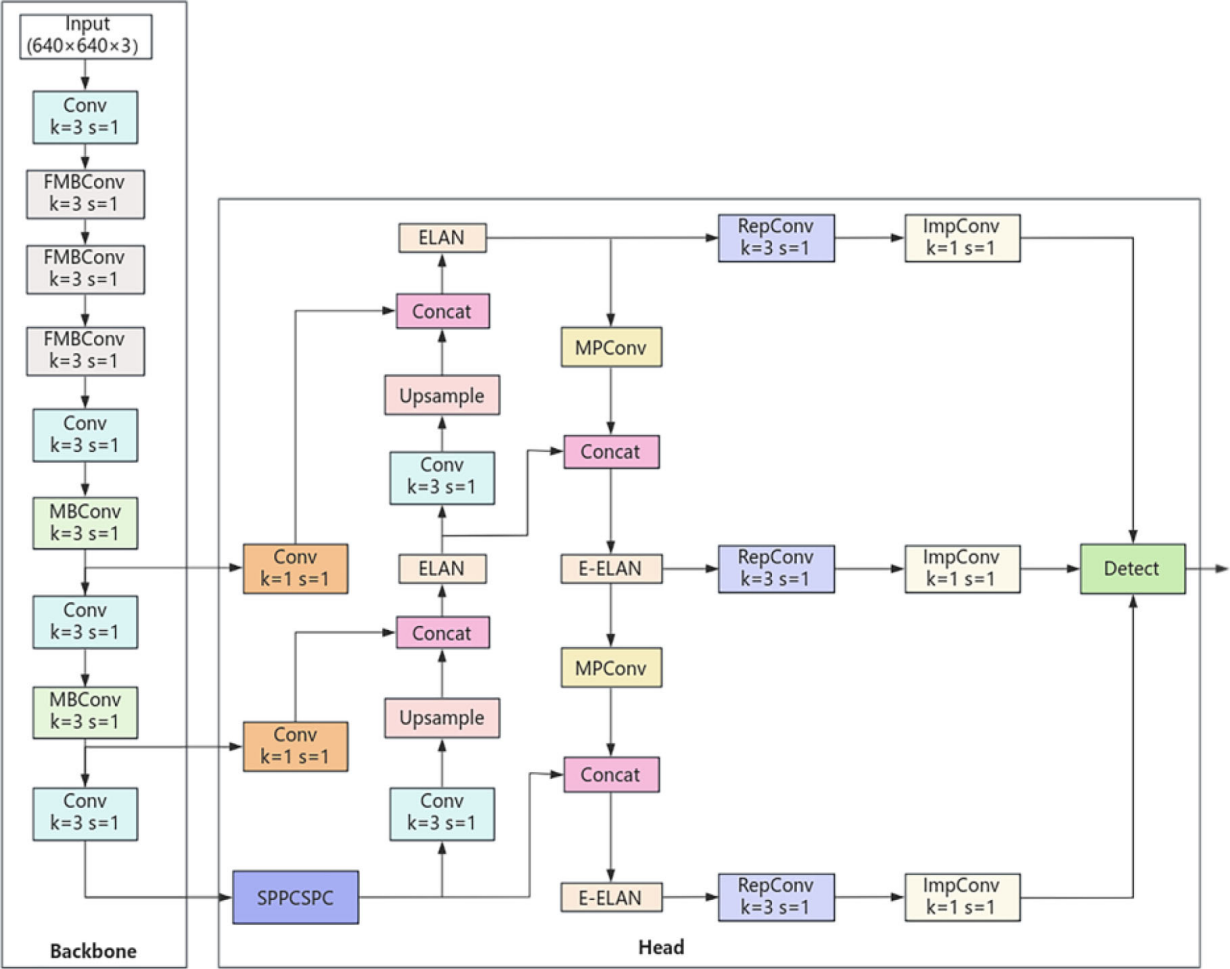
YOLOv7 network structure after changing the backbone to EfficientNetV2-S.

The YOLOv7 initially resized the input images to 640 × 640 size and fed them into the backbone network. Subsequently, the processed data passed through the head layer, resulting in the output of three different-sized feature maps. These maps then underwent Rep and Conv layers to produce predictive outcomes. The backbone network of YOLOv7 comprised a total of 50 layers, including 4 CBS layers followed by an ELAN layer. Following these are three MP + ELAN outputs corresponding to the outputs of C3/C4/C5, with dimensions of 80 * 80 * 512, 40 * 40 * 1024, and 20 * 20 * 1024, respectively. However, this sequential structure may incur information loss in transmission and demand more computational resources, which might not align with the requirements of field monitoring. Therefore, to meet the demands of outdoor monitoring and enhance detection accuracy and precision, this study opted for the lightweight EfficientNetV2-S to replace the backbone of the YOLOv7 model. This decision balances efficient parameter utilization while improving training speed (Liu et al., 2023). EfficientNetV2-S boasts enhanced feature extraction capabilities, supporting multi-scale feature extraction and better capturing image information to prevent the loss of small targets. Unlike the backbone of YOLOv7, EfficientNetV2-S employs a more efficient network structure, extracting more features by controlling the shortest and longest gradient paths while demonstrating stronger robustness. This design effectively reduces information loss during transmission, elevating the model’s performance (Tan and Le, 2021). Compared to a sequentially passed network structure, EfficientNetV2-S better retains and utilizes information, enhancing both accuracy and efficiency of the model. The modified YOLOv7 model is shown in **Figure 2**.

**Figure 3:**
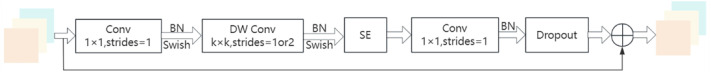
Structure of the MBConv module.

EfficientNetV2-S, is a deep neural network, which comprises several modules such as the Stem module, MobileNet Convolution (MBConv) module, Head module, and Classifier. The MBConv module serves as the core of EfficientNetV2-S and is known for its lightweight and efficiency (Shang et al., 2023), enabling them to conduct real-time monitoring in resource-constrained environments. This is crucial for field applications, particularly in mobile devices or embedded systems with limited computational resources. Designed to support multi-scale feature extraction, it better handles the diverse image data in outdoor environments. Such flexibility and efficiency make the MBConv module an ideal choice for meeting image processing demands in complex settings. The structure is illustrated in **Figure 3**.

**Figure 4:**
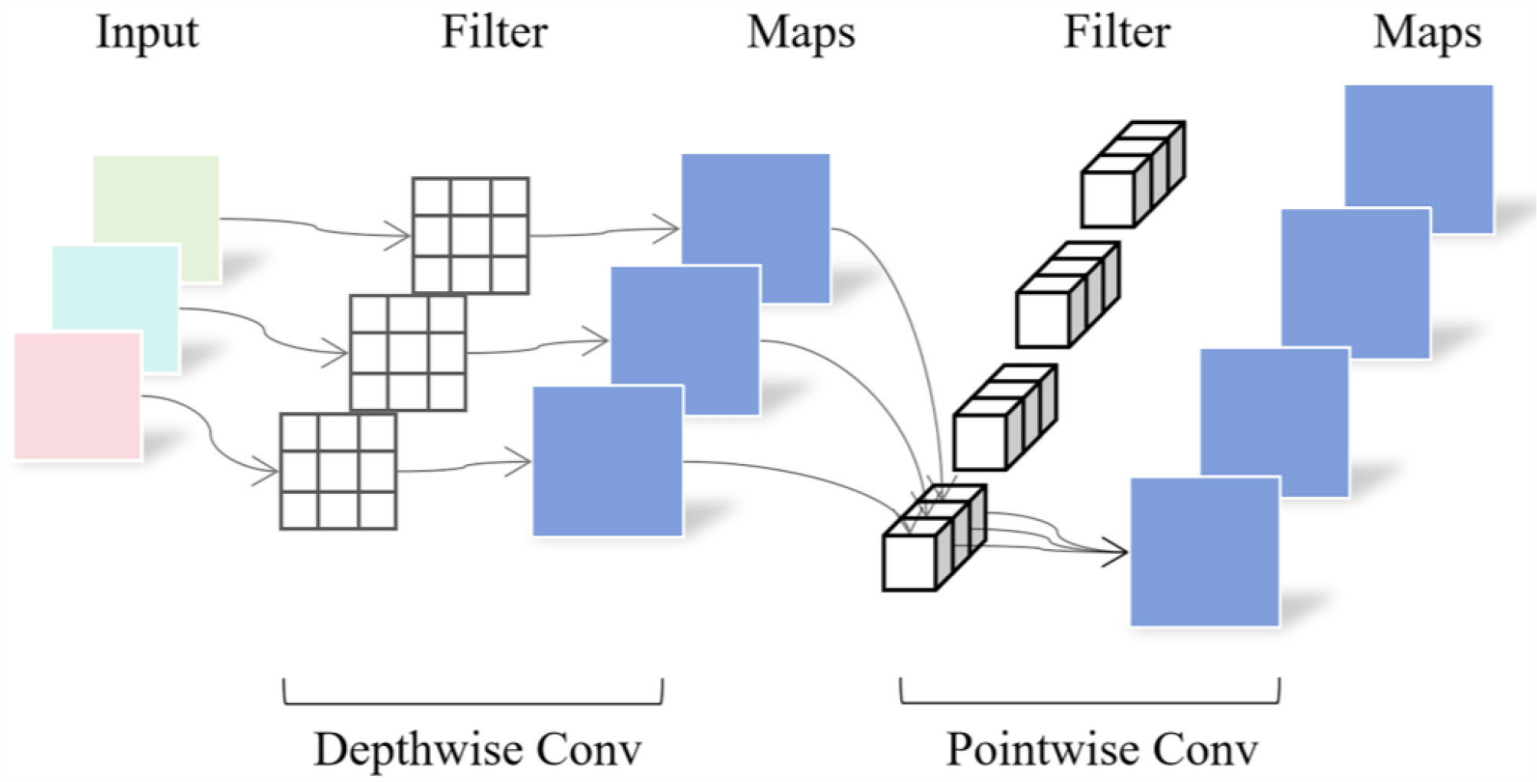
Structure of the DWConv module.

The Depthwise Separable Convolution (DWConv) module comprises two components: Depthwise Convolution and Pointwise Convolution. Depthwise Convolution operates independently on each input channel, capturing spatial information within feature maps effectively. Pointwise convolution mixes features across channels, generating final output feature maps. In natural environments, images often have complex backgrounds. DWConv helps capture local features, enhancing the model’s ability to generalize across different conditions, ensuring reliable operation in diverse environments. See **Figure 4** for the structure.

After inputting an image, Depthwise convolution independently conducts convolution operations on each input channel, significantly reducing the model’s parameter count. Additionally, Pointwise convolution, as a lightweight 1 x 1 convolution operation, effectively reduces computational costs while preserving spatial information within the feature maps. This combination enables DWConv to excel in lightweight model design and deployment on embedded devices, markedly enhancing computational efficiency and model performance while maintaining high feature extraction capabilities.

**Figure 5:**
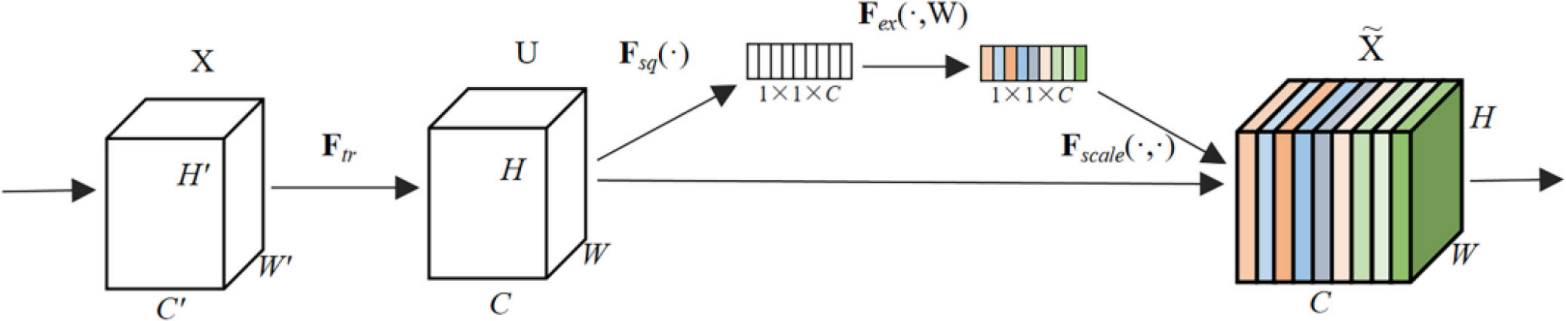
The flowchart of the SE algorithm.

The Squeeze-and-Excitation (SE) attention mechanism is a technique used to enhance the performance of deep neural networks in image recognition tasks. By dynamically adjusting the importance of each channel, it improves the network’s representation capabilities for different features. Within the MBConv, the SE mechanism adaptively learns channel weights, strengthening the network’s perception abilities for features at different scales and positions. This improves the model’s accuracy in identifying critical features, such as those present in images of pests such as *H. ligniperda*, and enhances performance across various image processing tasks. By highlighting prominent features in pest images, the SE mechanism enables more accurate identification and monitoring of pests. Additionally, it enhances the model’s robustness and generalization capabilities, allowing it to adapt to complex environmental factors. The flowchart of the SE algorithm is depicted in **Figure 5**.

The Transformation (F*_tr_*): The input feature map X comprises the height (*H*), width (*W*), and channels (*C*), encompassing raw image information the model processes. It undergoes transpose operations through feature mapping, generating the feature map U; Squeeze (F*_sq_*(·)): This operation globally averages pools the feature map, compressing its spatial dimensions into a 1×1×*C* vector, reducing the spatial size of the feature map; Excitation Function (F*_ex_*(·,W)): Represents the excitation function, generating a weight value for each feature channel; Scale Function (F*_scale_*(·,·)): Represents the scaling function, which weights the normalized weights obtained earlier onto each channel’s features; Finally, the weighted feature map is produced by multiplying the feature map with the obtained weights. Through this process, the model enhances the representation of crucial features, thereby improving its perception of key features and suppressing the influence of less important features.

**Figure 6:**
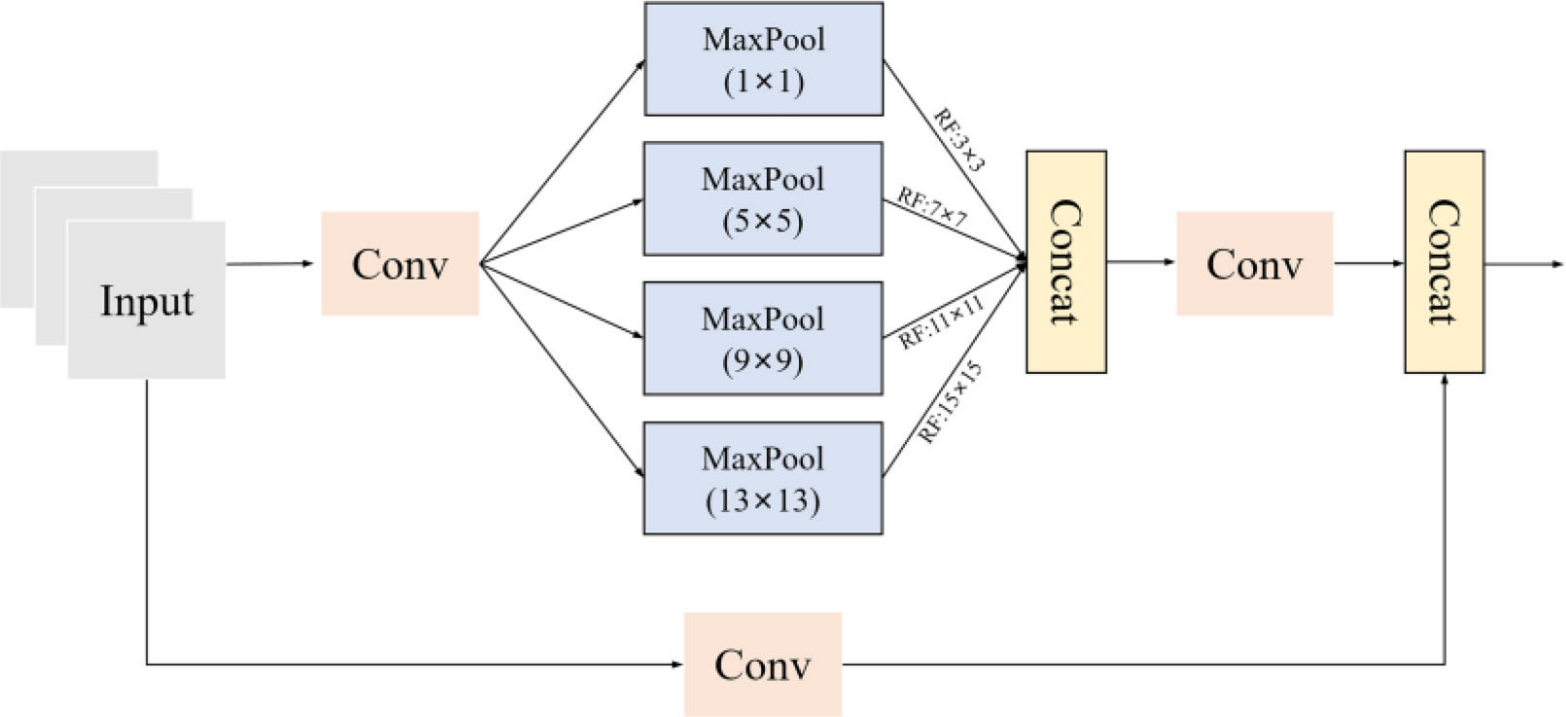
The diagram of the SPPCSPC structure involves convolutional operations followed by four types of pooling.

After extracting key features using the backbone, further optimization of feature extraction and information transmission is required. The feature map undergoes additional processing through the SPPCSPC structure. The SPPCSPC module in YOLOv7 combines Spatial Pyramid Pooling (SPP) and Cross Stage Partial Connections (CSP). SPP utilizes spatial pyramid pooling, extracting features at various scales without altering the feature map size. This allows the model to capture features across different spatial scales, enhancing its perception of multi-scale targets. By aggregating information at different scales, it augments the model’s understanding of the background around targets, contributing to improved detection accuracy of small-sized objects in complex environments. CSP, on the other hand, employs cross-stage partial connections to share information between each stage, reducing computational load and alleviating gradient vanishing issues in deep network structures. It enhances feature transmission efficiency and robustness of the model to variations in *H. ligniperda* habitats, enabling the model to better adapt to complex natural environments. The SPP layer is utilized to extract features at various scales, the CSP connection layer shares information, while the convolutional layers further extract features. This architecture effectively enhances network performance and achieves improved detection accuracy while maintaining a relatively lower computational load. Monitoring *H. ligniperda* in the wild, particularly through real-time surveillance in dynamic natural environments, holds significant practical significance. This aids in learning to recognize small-sized targets like *H. ligniperda* amidst complex backgrounds. The SPPCSPC structure is depicted in **Figure 6**.

### 2.5 The loss function of YOLOv7

The Loss Function of YOLOv7 typically comprises multiple components, primarily the Classification Loss, Localization Loss, and Confidence Loss.

The Classification Loss measures the model’s accuracy in classifying object categories. It typically utilizes Cross-Entropy Loss or Binary Cross-Entropy Loss. The formula is represented as Equation 1.

(1) $$L_{class}=-\frac{1}{N}\sum_{i=1}^{N}\sum_{c=1}^{C}y_{i,c}\log({\hat{y}}_{i,c})$$

Where *L*_class_ represents the Classification Loss, *N* denotes the sample count, *C* is the number of classes, *y_i,c_*, is the true label for sample *i* in class *c*, and *ŷ_i,c_* is the predicted probability by the model for class *c* of sample *i*.

(2) $$L_{loc}=\lambda_{coord}\sum_{i=1}^{N}\left({\left(x_{i}-{\hat{x}}_{i}\right)}^{2}+{\left(y_{i}-{\hat{y}}_{i}\right)}^{2}\right)+\lambda_{\dim}\sum_{i=1}^{N}\left({\left(w_{i}-{\hat{w}}_{i}\right)}^{2}+{\left(h_{i}-{\hat{h}}_{ï}\right)}^{2}\right)$$

The Localization Loss measures the model’s accuracy in predicting the target’s position (bounding box coordinates). The formula is depicted as Equation 2.

Where *L*_loc_ represents the Localization Loss, *N* is the sample count, *x_i_*, *y_i_*, *w_i_*, *h_i_* denotes the true center coordinates and dimensions of the bounding box for sample *i*. $\hat{x}i$, $\hat{y}i$, $\hat{w}i$, $\hat{h}i$, represents the predicted center coordinates and dimensions of the bounding box by the model for sample *i*, and λ_coord_ and λ_dim_ signifies the weight of the Localization Loss.

The Confidence Loss measures the model’s accuracy in predicting the confidence of the target’s presence (indicating whether an object is detected). The formula is shown as Equation 3.

(3) $$L_{conf}=\sum_{i=1}^{N}{\left(C_{i}-{\hat{C}}_{i}\right)}^{2}$$

Where *L*_conf_ represents the Confidence Loss, *N* is the sample count, *C_i_* denotes the true confidence of the sample (1 for containing the object, 0 for not containing), and *Ĉ _i_* represents the model’s predicted confidence for sample *i*.

Improving the loss function with the concept of Focal Loss helps focus the model’s attention on the correct feature sets within the data, leading to enhanced and faster convergence for acquiring the most suitable model for target samples. Such enhancements aid in improving the model’s ability to recognize critical features, intensifying the model’s focus on crucial targets, consequently elevating overall detection and recognition accuracy.

### 2.6 Model evaluation metrics. Evaluation metrics equation the model

To validate the superior performance of the enhanced YOLOv7 model, key metrics such as Precision, AP, mAP, Recall, and others can be utilized. Equations 4–6 are as follows:

(4) $$Precison=\frac{TP}{TP+FP}$$

(5) $$Recall=\frac{TP}{TP+FN}$$

(6) $$mAP=\frac{1}{N}\sum_{i=1}^{N}\int_{0}^{1}PrecisiondRecall\;$$

Where: True Positives (*TP*) are the samples predicted as positive and are actually positive instances; False Positives (*FP*) are the samples predicted as positive but are actually negative instances; False Negatives (*FN*) are the samples predicted as negative but are actually positive instances; and *N* represents the number of sample categories.

## 3 Experimental result and analyses

### 3.1 Experimental platform and parameters

The establishment and progression of deep learning necessitate the meticulous configuration of an appropriate environment conducive to its growth. Such a meticulously curated environment significantly influences training speed and overall performance. The specifics of the parameters employed in this experiment have been delineated in **Table 1**.

**Table 1:** The configuration of the deep learning environment and training hyperparameters.

Parameter	Value
Operating System	Windows11
CPU	AMD Ryzen 9 7945HX with Radeon Graphics 2.50 GHz
GPU	NVIDIA GeForce RTX 4060 (8GB)
RAM	32GB
Framework	Pytorch1.7.1 (CUDA11.0)
Momentum	0.937
Initial Learning Rate	0.01
Batch Size	16
Weight Decay	0.0005
Total Epochs	300

### 3.2 The focal loss optimization function

The Focal Loss is a type of loss function specifically designed to address class imbalance. Due to the relatively small area occupied by *H. ligniperda* in images, its representation on feature maps might not be distinct. Focal Loss enhances the model’s focus on hard-to-classify samples, thereby improving the model’s capability to detect small objects. In the target detection task of this experiment, the number of samples for actual background categories in the wild was typically much larger than the number of samples for the target category. This resulted in a class imbalance. Traditional cross-entropy loss functions might cause the model to overly focus on the more abundant background categories while neglecting the less frequent target categories in such scenarios. Focal Loss addresses this issue by introducing a modulating factor. The formula is depicted as Equation 7.

(7) $$FL\left(p_{t}\right)=-{\left(1-p_{t}\right)}^{\gamma }\text{log}\left(p_{t}\right)$$

**Figure 7:**
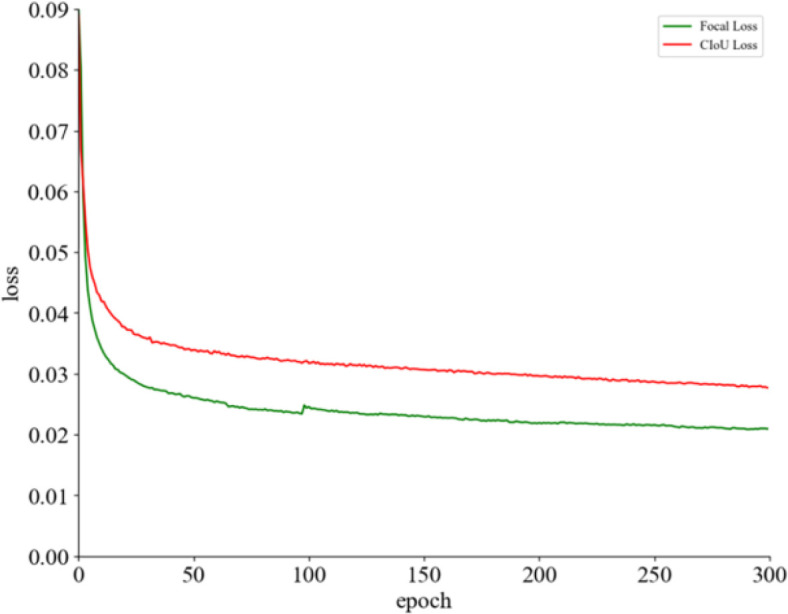
Comparison chart of loss functions.

Where *p_t_* represents the predicted probability of the correct class by the model, γ is a hyperparameter used to control the shape of the modulating factor. When a sample is easily classified correctly (i.e., *p_t_* approaches 1), (1 - pt) ^γ^ approaches 0, thereby reducing its contribution to the total loss. Conversely, for samples that are harder to classify correctly (i.e., *p_t_*) approaches 0), (1 - pt) ^γ^ approaches 1, resulting in a larger weight for these samples in the total loss. The comparison of loss function improvements before and after is illustrated in **Figure 7**.

**Figure 8:**
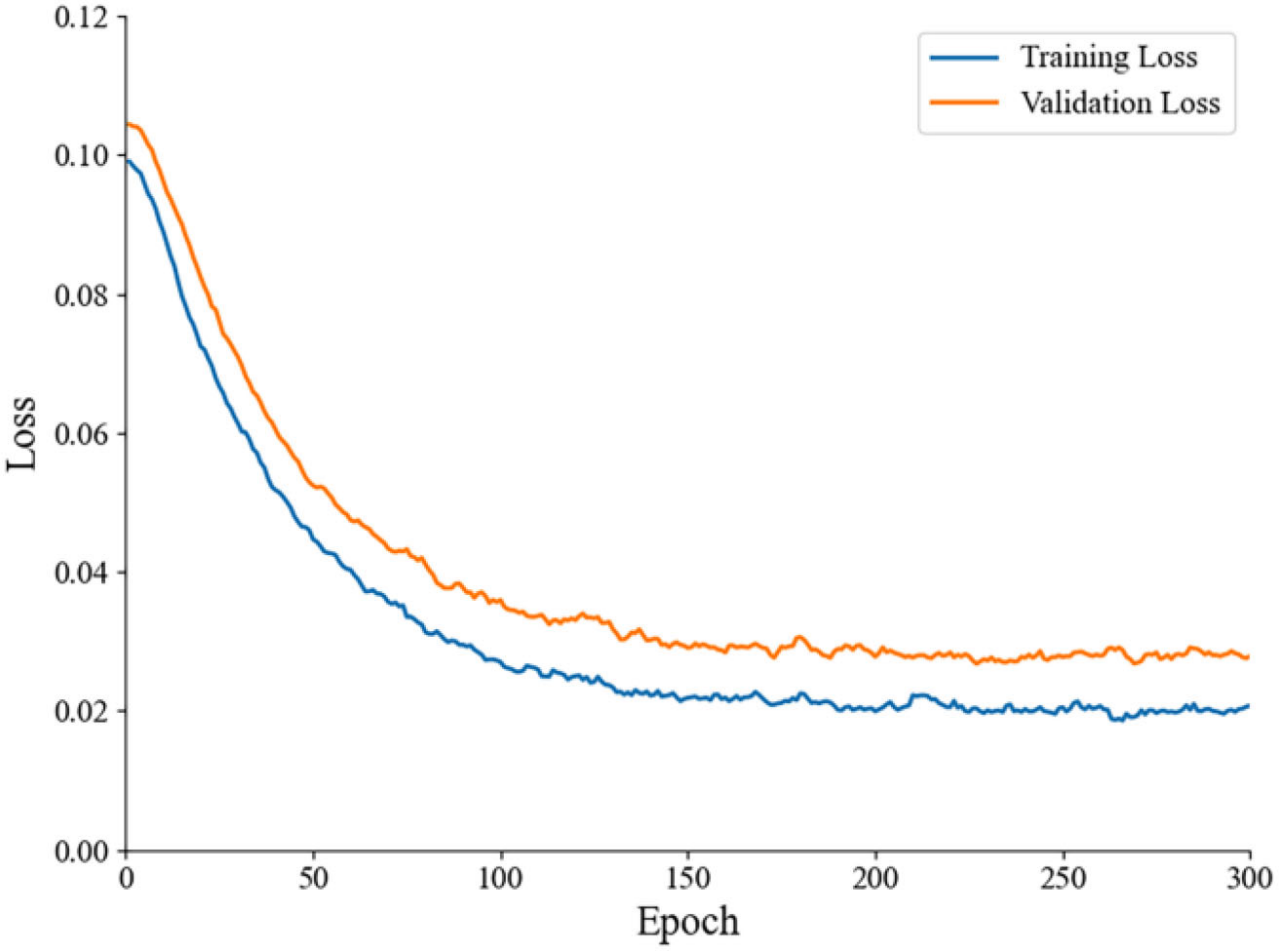
Comparison of training loss and validation loss curves across epochs.

To further evaluate the generalization ability of the model and rule out overfitting, we monitored the training and validation losses throughout the training process. **Figure 8** illustrates the comparison between the training loss and validation loss curves across 300 epochs. As observed, both curves show a steady decreasing trend and converge to a stable value. Importantly, the validation loss closely follows the training loss without exhibiting any significant divergence or upward trend in the later stages. This indicates that the improved YOLOv7 model generalizes well to unseen data and does not suffer from severe overfitting.

### 3.3 Comparison of results from mainstream recognition models

To verify the effectiveness of each improvement strategy proposed in this paper, we conducted an ablation study. We compared the performance of the original YOLOv7 baseline, the model with only the EfficientNetV2-S backbone, the model with only the Focal Loss function, and our final proposed model (combining both). The results are presented in **Table 2**.

**Table 2:** Ablation study of different improvement strategies.

Model	Backbone	Loss function	mAP@0.5	Param
Baseline	YOLOv7	CIoU	78.1%	143MB
Method A	EfficientNetV2-S	CIoU	80.4%	70MB
Method B	YOLOv7	Focal Loss	79.2%	143MB
Ours	EfficientNetV2-S	Focal Loss	82.5%	70MB

As shown in the table, replacing the original backbone with EfficientNetV2-S (Method A) not only reduced the model size significantly from 143 MB to 70 MB, achieving lightweight deployment, but also improved the mAP by 2.3% (from 78.1% to 80.4%). This indicates that EfficientNetV2-S extracts more robust features for small pest targets. Furthermore, applying Focal Loss alone (Method B) improved the mAP to 79.2%, proving its effectiveness in addressing class imbalance. Finally, our proposed method, which integrates both improvements, achieved the best performance with an mAP of 82.5%, demonstrating that the combination of a lightweight backbone and an optimized loss function yields the optimal balance between accuracy and efficiency.

To strictly validate our method, we introduced the YOLOv8 for comparison. While YOLOv8 shows a slightly higher Recall (81.2%), our Improved YOLOv7+ achieves superior Precision (83.4%) and localization accuracy (mAP@0.5:0.95 of 63.2%), effectively reducing false positives in complex trap backgrounds. Crucially for embedded forestry applications, our model is 16.7% smaller (70 MB vs. 84 MB) and faster than YOLOv8, proving it to be the more efficient choice for resource-constrained edge devices, like Raspberry Pi. The results are presented in **Table 3**.

**Table 3:** The performance comparison of target detection algorithms.

Model	Params	Precision	Recall	mAP@0.5	mAP@0.5:0.95	FPS
Faster R-CNN	162MB	59.3%	65.4%	60.2%	32.5%	31
YOLOv5	89MB	73.9%	77.8%	75.6%	47.2%	45
YOLOv7	143MB	80.2%	73.1%	78.1%	54.3%	49
YOLOv8	84MB	82.1%	81.2%	82.9%	61.8%	55
YOLOv7+	70MB	83.4%	80.6%	82.5%	63.2%	57

**Figure 9:**
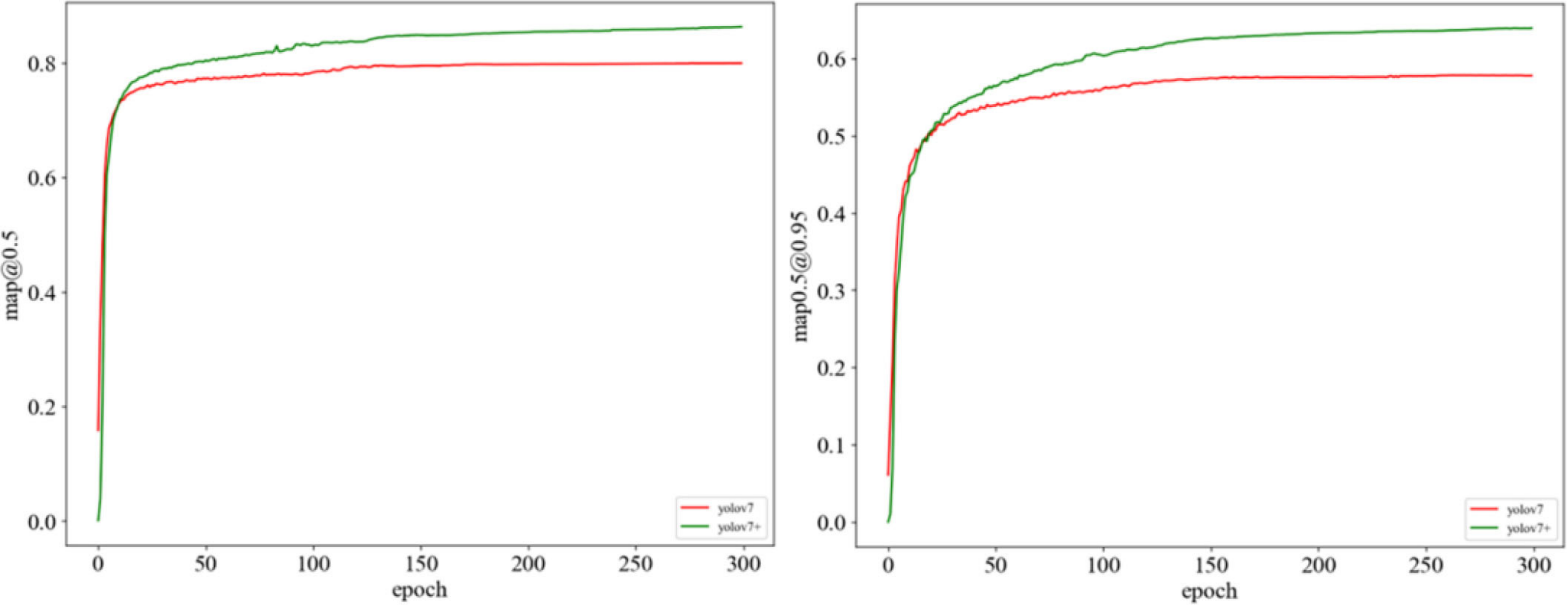
Comparison of mAP convergence curves between YOLOv7 and YOLOv7+.

mAP@0.5 and mAP@0.5:0.95 provide different perspectives on model performance. mAP@0.5 fixes the IoU threshold at 0.5, emphasizing the model’s performance at lenient IoU thresholds. On the other hand, mAP@0.5:0.95 considers various thresholds (0.5, 0.55, 0.6, 0.65, 0.7, 0.75, 0.8, 0.85, 0.9, 0.95), offering a comprehensive evaluation of the model’s performance across different strictness levels. **Figure 9** illustrates that our model demonstrated commendable performance.

The aforementioned data demonstrated the effective enhancement of *H. ligniperda* detection model within bait traps through the adoption of the new backbone network, EfficientNetV2-S, and the refinement of the loss function using Focal Loss principles.

**Figure 10:**
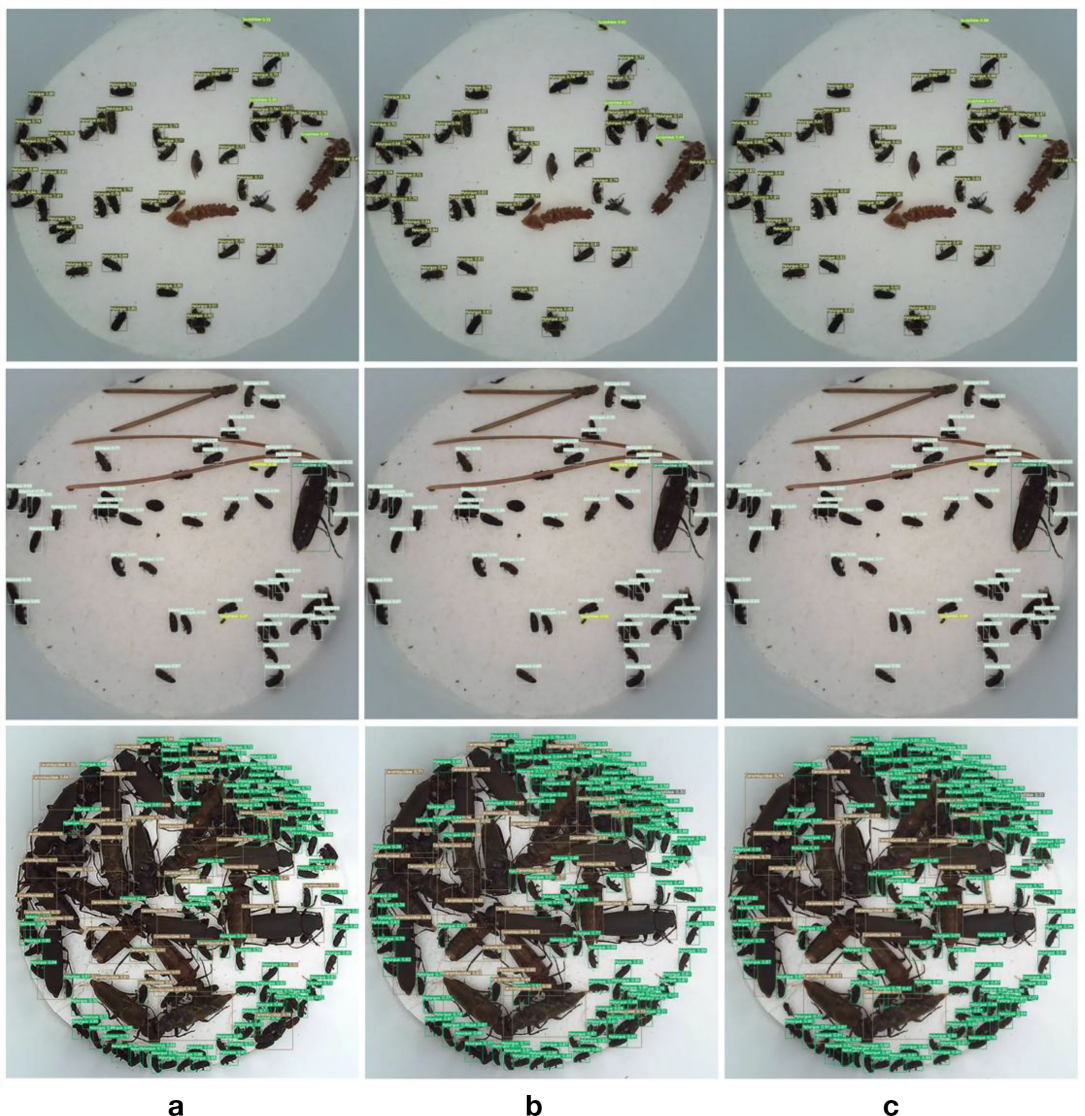
The comparison of results: **(a)** YOLOv5 recognition image; **(b)** YOLOv7 recognition image; **(c)** Enhanced model recognition image.

**Figure 9** validates the detection capabilities of this model in actual bait trap environments and presents them directly. In **Figure 10**, the first column represents the YOLOv5 recognition result, the second column shows the YOLOv7 recognition result, and the third column depicts the improved model’s recognition result. It can be observed that the recognition accuracy of the first two models is significantly lower than that of the improved model, and instances of both missed detections and false positives are evident. Moreover, in the third-row image where occlusion is substantial, our model achieved satisfactory results by successfully identifying all targets. This demonstrates the superior precision and accuracy of this model.

## 4 Discussions

The forest pest, *Hylurgus ligniperda* (Fabricius) poses a significant threat to forestry, causing substantial damage and economic losses due to its large population and rapid spread. Accurate and timely detection of this pest is crucial for effective pest management in forestry. Timely and precise monitoring enables prompt responses to pest outbreaks, minimizing losses and conserving resources. This study presents substantial progress in real-time detection of *H. ligniperda* in forest environments through the enhanced YOLOv7+ method. It achieved a balance between detection accuracy and efficiency. Specifically, compared to the latest YOLOv8, our model demonstrates superior precision and localization accuracy (mAP@0.5:0.95) while maintaining a smaller parameter size, making it more suitable for resource-constrained embedded devices. Furthermore, the training and validation loss analysis (**Figure 8**) confirms that our model possesses strong generalization capabilities and does not suffer from overfitting, ensuring reliable performance in diverse field conditions.

In our future research, we aim to deploy the improved YOLOv7+ model on mobile devices for real-time detection of small-target pests in forest environments, aiming to optimize monitoring costs while enhancing early warning capabilities for forest pest outbreaks. Integrating this method into the Internet of Things (IoT) framework will aid in early pest detection and mitigation strategies, mitigating economic losses. Nevertheless, the limited computational resources and storage space of mobile devices pose challenges to ensuring real-time pest detection and maintaining data transmission and processing capabilities within IoT connections. Our next research phase will focus on optimizing both the model and hardware processing to achieve a balance in performance under these constraints. The realization of this research holds significant implications for the intelligent management of forestry resources.

## 5 Conclusions

In this study, we proposed an enhanced YOLOv7 deep learning model based on improvements made within the natural environment of insect traps, aiming for rapid and precise detection of a crucial forestry pest, *Hylurgus ligniperda* (Fabricius). This approach employed the lighter EfficientNetV2-S as the backbone network for the new model, ensuring detection accuracy and precision while maintaining a lightweight structure. With the integrated SE attention mechanism, the model learned to recognize critical features of the target, enhancing its ability to perceive features across different scales and spatial positions. Furthermore, we optimized the entire model using the Focal Loss concept, particularly focusing on challenging-to-classify samples, addressing class imbalance issues, and improving detection and recognition capabilities for small targets.

Experimental tests demonstrated significant improvements of the refined YOLOv7 over both YOLOv5 and the original YOLOv7. The method achieved a precision rate of 83.4% and a recall rate of 80.6%. Compared to Faster R-CNN, YOLOv5, the initial YOLOv7, and even the state-of-the-art YOLOv8, this model attained the highest mAP value (82.5%) and mAP@0.5:0.95 on the established dataset, showcasing enhanced robustness and superior localization capabilities Therefore, the refined YOLOv7 exhibited greater precision in recognizing targets. Furthermore, in contrast to the original YOLOv7, the model demonstrates a substantial reduction in size, facilitating the deployment of deep learning models on embedded and mobile devices for timely pest monitoring.

In the future, our goal is to integrate our research model into mobile terminal devices to achieve real-time detection and analysis of forest pests. We will continuously improve the model and fine-tune the hardware to adapt to different scenarios, thereby enhancing the model’s applicability in various forest environments and with different pests. Overall, the enhanced YOLOv7+ holds vast potential for applications in forestry pest management, providing effective support for forestry workers.

## Data Availability

The raw data supporting the conclusions of this article will be made available by the authors, without undue reservation.
